# Cloacal Microbiome Structure in a Long-Distance Migratory Bird Assessed Using Deep 16sRNA Pyrosequencing

**DOI:** 10.1371/journal.pone.0137401

**Published:** 2015-09-11

**Authors:** Jakub Kreisinger, Dagmar Čížková, Lucie Kropáčková, Tomáš Albrecht

**Affiliations:** 1 Studenec Research Facility, Institute of Vertebrate Biology, Academy of Sciences of the Czech Republic, Květná 8, 603 65 Brno, Czech Republic; 2 Department of Zoology, Faculty of Science, Charles University Prague, Viničná 7, 128 44 Prague 2, Czech Republic; 3 Department of Biodiversity and Molecular Ecology, Fondazione Edmund Mach, Research and Innovation Centre, I-38010 San Michele all’Adige, TN, Italy; Università degli Studi di Milano-Bicocca, ITALY

## Abstract

Effects of vertebrate-associated microbiota on physiology and health are of significant interest in current biological research. Most previous studies have focused on host-microbiota interactions in captive-bred mammalian models. These interactions and their outcomes are still relatively understudied, however, in wild populations and non-mammalian taxa. Using deep pyrosequencing, we described the cloacal microbiome (CM) composition in free living barn swallows *Hirundo rustica*, a long-distance migratory passerine bird. Barn swallow CM was dominated by bacteria of the *Actinobacteria*, *Proteobacteria* and *Firmicutes* phyla. *Bacteroidetes*, which represent an important proportion of the digestive tract microbiome in many vertebrate species, was relatively rare in barn swallow CM (< 5%). CM composition did not differ between males and females. A significant correlation of CM within breeding pair members is consistent with the hypothesis that cloacal contact during within-pair copulation may promote transfer of bacterial assemblages. This effect on CM composition had a relatively low effect size, however, possibly due to the species’ high level of sexual promiscuity.

## Introduction

Vertebrate digestive tracts are inhabited by a taxonomically and functionally diverse community of bacteria, usually dominated by obligatory anaerobes [1,2]. Indeed, the cell and active gene count of this community may exceed that of the host genome by at least one order of magnitude [3]. Hence, it is no surprise that gastrointestinal tract microbiota (GTM) interact with a broad range of host physiological systems and provide ecosystem services of considerable value. In particular, GTM affect metabolism efficiency [4,5], modulate the host’s immune system [6], play a significant role in defence against pathogens [7,8] and enable synthesis of substances that cannot be synthesised by enzymes encoded by the host’s genome [9,10]. GTM dysbiosis is often associated with metabolic [11,12], autoimmune [13] and neurological disorders [10,14] and can also increase the risk of pathogen invasion [7,8].

Recent advances in parallel high-throughput sequencing have enabled detailed insights into the complex interplay between GTM and vertebrate physiological status [5,11]. To date, most of this research has been focused on biomedical aspects of host/GTM interactions in humans and captive-bred mammalian model species [3,4,11,15]. The effect of GTM on host physiology also has relevance to ecological and evolutionary studies of wild populations. GTM composition has been shown to be associated with mate choice [16], for example, including propensity for within- and extra-pair copulations [17,18]. There is also evidence that social contact mediates horizontal transfer of GTM from parents to progeny [19–21], between sexual partners [22,23] or between members of a social community [7,24]. This transfer can have a long-lasting effect on fitness-related traits such as metabolism efficiency or pathogenesis susceptibility. Despite its potential importance, the current low knowledge level on GTM composition in free-living non-mammalian vertebrates and on factors shaping intra- and inter-specific variation, but see [21,25,26] precludes any general conclusions.

In this study, we focus on cloacal microbiome (CM) composition in a free-living population of barn swallows (*Hirundo rustica*), an insectivorous long-distance migratory passerine bird. The barn swallow is a traditional model species for research into reproductive biology and evolutionary ecology, and especially for studies of sexual selection and sperm competition [27–29]. To date, there has been no attempt to extend this research by including information on GTM composition, despite it having particular relevance in these fields.

Biogeographically, e.g. [1], CM is a subset of GTM colonising the distal part of the gut communicating directly with the urogenital tract and the external environment. Factors associated with inter-individual CM variation in wild bird populations have already received some attention, particularly as regards to horizontal transfer of CM from parents to progeny [21] or between sexual partners during copulation [25,30,31]. Many of these studies used cultivation based methods that only capture a low proportion of total CM, e.g. [32]. A few studies have used culture independent methods, such as Automated rRNA Intergenic Spacer Analysis (ARISA), Denaturing Gradient Gel Electrophoresis (DGGE) or cloning and clone sequencing of 16s rRNA amplicons [25,33] however, these approaches are also likely to suffer from compromised CM coverage and taxonomic resolution.

In order to analyse barn swallow CM composition, we applied 454 pyrosequencing of 16s rRNA amplicons. The resulting data were used to assess whether sex, breeding pair identity or colony identity influenced inter-individual variation in microbiota composition during the breeding season.

## Methods

### Field sampling

We sampled CM from seven barn swallow breeding pairs (i.e. seven males and seven females) from two colonies, each around 4.5 km from the village of Lužnice in the Czech Republic (49°3'56.90"N, 14°45'20.38"E). Both colonies (ca. 40 breeding pairs at each locality, hereafter”Kotrbů” and “Šaloun”) were located in small livestock farms. The composition of livestock differed between these two localities. Cattle and pigs dominated in Kotrbů, whereas sheep and goats were more common in Šaloun. CM sampling was performed during the nestling period (second breeding attempt, late June). We assume that the last within-pair copulations occurred approx. 2–3 weeks before the data collection—given that within-pair copulations occur mostly during or before egg laying and the length of incubation period is 12–18 days in this species [34]. The CM was collected using sterile DNA-free microbiological nylon swabs (minitip FLOQSwabs, Copan, Italy) inserted ca. 10 mm inside the cloaca for approx. 20 seconds and gently twisted by approx. 360 degrees. These samples were then stored in 2 ml DNA-free microcentrifuge tubes (Simport, Canada) at -80°C until sample processing, which was performed within one month of sample collection. The samples were collected over three consecutive days in order to minimize the probability that observed inter-individual variation was biased by temporal fluctuations in CM composition.

All field procedures were approved by the Animal Care and Use Committees at the Czech Academy of Sciences (041/2011), and Charles University in Prague (4789/2008-30). Owners of farms, where we collected samples, gave us the permission to conduct this work.

### Pyrosequencing

DNA was extracted in a sterile laminar flow cabinet using the Qiagen Stool kit (Qiagen, Germany). Bacterial barcoding was performed using the universal primers MPRK341F (CCTAYGGGRBGCASCAG) and MPRK806R (GGACTACNNGGGTATCTAAT) that amplify the ~466 bp fragment, including the V3 and V4 regions of *Escherichia coli* 16S rDNA [35]. Sequences of these primers were included in fusion primers used to perform polymerase chain reactions (PCR). Forward fusion primers, represented by adaptor B sequence (Lib A), the unique tag sequence from the Roche MID library and the MPRK341F primer sequence, differed between individuals sequenced. The reverse fusion primer consisted of the Titanium adaptor A sequence (Lib A) and the MPRK806R primer sequence.

PCR was performed using a 30 μl solution consisting of 1x Qiagen Multiplex PCR Master Mix (Qiagen, Germany), forward and reverse fusion primers at final concentration 0.5 μM, and 8 μl of DNA solution. PCR conditions were as follows: initial denaturation at 95°C for 15 min; followed by 35 cycles of 94°C (30 sec), 56°C (90 sec), 72°C (60 sec); and a final extension at 72°C (20 min). PCR products were incubated at 70°C for three minutes and then stored on ice. The samples were then run on 1% agarose gel and bands of appropriate size were excised from the gel and purified using the QIAquick gel extraction kit (Qiagen, Germany) according to the manufacturer’s instructions using 30 μl of buffer in the elution step. Concentration of the purified PCR product was measured using a Qubit fluorometer (Invitrogen, USA) and normalised. Pyrosequencing was performed via a single run on a GS Junior sequencer (ROCHE, Switzerland) using Titanium chemistry according to the manufacturer’s instructions. Demultiplexed sff files have been deposited in the European Nucleotide Archive: http://www.ebi.ac.uk/ena/data/view/PRJEB7057.

### Analysis of 454 data

Sequences with low quality scores (average quality score < 0.25), that included more than three ambiguously determined nucleotides, that were shorter than 200bp, or that did not perfectly match forward primer sequences or tags were excluded from further analysis. Mid- and primer regions were trimmed using *QIIME 1*.*8*.*0* [36] and the resulting fasta file was denoised using the *Acacia* software [37], while chimeric sequences were identified and filtered out using *USEARCH* [38]. As recommended by May et al. [39], the *TBC* algorithm [40] was used to cluster the resulting high quality sequences into operational taxonomic units (OTUs) with a 97% similarity threshold. *TBC* output was subsequently parsed using custom *R* [41] and UNIX scripts to produce a *QIIME* formatted OTU table (presenting the sequence count for OTUs in individual samples).

Taxonomy of representative sequence for OTUs was assigned using *RDP classifier* [42], with a posterior confidence level of > 0.80. Representative sequences were aligned using *PyNast* and Greengenes Core Set Alignment [43] and a minimum evolution phylogenetic tree was constructed based on the procedure implemented in *FastTree* [44]. Hellinger distances between samples were calculated based on OTU abundance data. In addition, the phylogenetic tree, together with data on OTU abundances, was used to calculate both unweighted and weighted UniFrac distances [45] between samples. To avoid potential bias associated with unequal sequencing depth, distances were calculated based on a random subsample corresponding to 1600 reads (i.e. approximate minimum achieved sequencing depth) per individual.

The Chao1 index [46], phylogenetic diversity index (computed as total branch length), and number of OTUs detected in individual samples were calculated to provide further information on CM alpha diversity. In addition, total OTU richness for individual samples was estimated based on best-fitting parametric model implemented in *CatchAll* [47]. Coverage of CM diversity by our sequencing data was assessed based on rarefaction analysis and Goods coverage index [48]. Paired t-tests were used to test whether alpha diversity differed between males and females. Distances between samples were visualised using non-metric multidimensional scaling (NMDS) and distance-based redundancy analysis (db-RDA), implemented in the *vegan* package [49], was used to test whether CM composition differed systematically between males and females and between breeding colonies. The betadisper function (analogous to Levene’s test of equality of variance), was used as a next step to assess whether inter-individual variation in Hellinger and UniFrac distance differed between males and females. Finally, we applied the Wilcoxon signed rank test to detect differences in proportional composition of individual bacterial phyla and families between males and females. The same approach was used to compare the proportion of individual OTUs (i.e. number of reads for a given OTU in a given sample divided by total number of reads for a given sample) that were represented by < 0.1% reads (number of OTUs = 123, see S2 Table for more detail). The q-value method was applied to account for false discoveries when using multiple comparisons [50] (q-value threshold was set to 0.05). In addition, corrected moment estimates of k parameter of the negative binomial distribution was calculated for these OTUs. This index is widely used in parasitology to quantify the level of parasite aggregation among hosts. Low values of this index imply highly aggregated distribution, whereas high values (k > 20) indicate near-Poisson distribution of infection intensities [51].

Two analytical approaches were applied to test whether individuals from the same breeding pair exhibited a higher level of similarity than expected by chance. First, we compared the observed mean of within-pair distances (Hellinger and both weighted and unweighted UniFrac) with the null distribution of mean distances for randomly paired males and females (n = 1000 randomly generated pairs). This individual-centred approach is highly conservative due to the relatively low sample size of our study. Second, an OTU-centred resampling approach was used to assess whether relative abundances of individual OTUs were non-randomly correlated between males and females within individual breeding pairs. This analysis was run on a subset of 153 OTUs occurring in ≥ 4 individuals and including 77% of the original high quality reads. Within-pair correlation of each OTU proportion was assessed using Spearman’s correlation coefficient (Spearman’s r); the Fisher’s z-transformed mean being used as the within-pair similarity index. In the next step, randomized matrices (n = 1000) were constructed by reshuffling the individual identity in the original matrix of OTU proportions for individual samples which at the same time accounted for sex identity (i.e. randomly selected males was paired with randomly selected females). Mean Fisher’s z-transformed Spearman’s r was computed for each randomised matrix, as described above, and the resulting null distribution was used to assess statistical significance of within-pair community correlation. The outcome of these analyses were also expressed as community-specific standardised effect sizes (SES) using the formula *(CORor–mean CORsim)/sdCORsim* [52], where *CORor* is the mean of Fisher’s z transformed correlations within actual pairs, mean *CORsim* is the mean Fisher’s z transformed Spearman’s r for randomised matrices, and *sdCORsim* is its standard deviation. We tested this approach using different OTU filtering criteria, Pearson correlations and raw instead of Fisher’s z-transformation correlation coefficients, and found that the results of these analyses remained unchanged.

All statistical analyses were performed in *R 3*.*1*.*0* [53], the statistical significance for all tests being two-tailed. The ‘*phyloseq*’ package in *R* [41] was used for filtering and manipulating community data.

## Results

**Fig 1:**
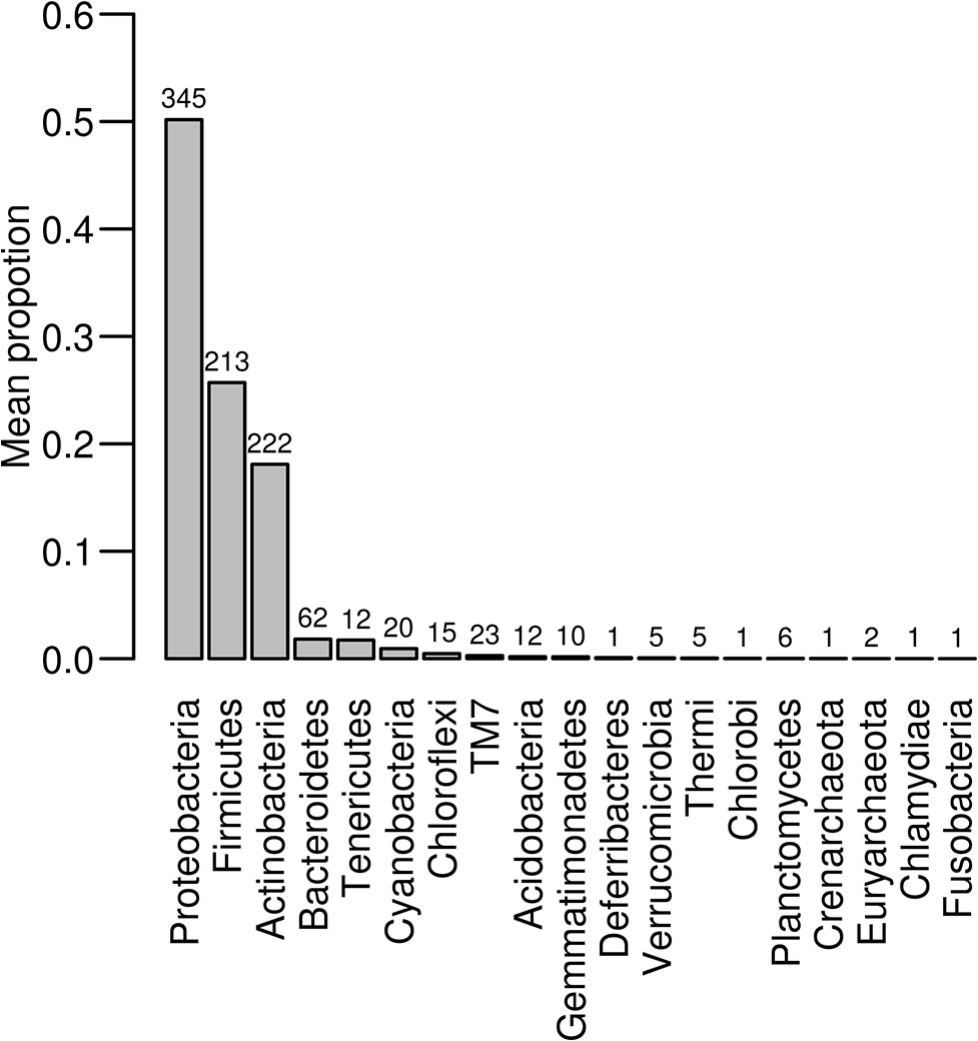
Taxonomic composition of barn swallow cloacal microbiome. Bar heights correspond to the proportion of sequences assigned to individual bacterial phyla. Numbers above the bars indicate number of 97% TBC OTUs corresponding to a given phylum.

We analysed 71,100 sequences that passed quality filtering and were not chimeric, with the number of high quality sequences ranging between 1,656 and 8,110 (mean = 5,078) per sample. Sequences were clustered in 981 OTUs (754 non-singleton; details in S1 Table). The Goods coverage index ranged between 0.975 and 0.998 (mean = 0.992). This, along with the results of rarefaction analysis (presented in S1 Fig), suggest that the sequencing depth in our study was sufficient to capture the majority of CM alpha diversity. Based on taxonomic assignation, bacteria from the phyla *Proteobacteria*, *Firmucutes* and *Actinobacteria* dominated the CM. We further recorded members of 17 other bacterial phyla and two archaebacterial phyla (*Crenarchaeota* in one OTU and *Euryarchaeota* in two OTUs) at low frequencies (Fig 1; see S2 Fig for more details on taxonomic classification). The level of inter-individual variation in CM composition was pronounced as just four OTUs were detected in all samples and only 52 OTUs in more than 50% of individuals.

**Fig 2:**
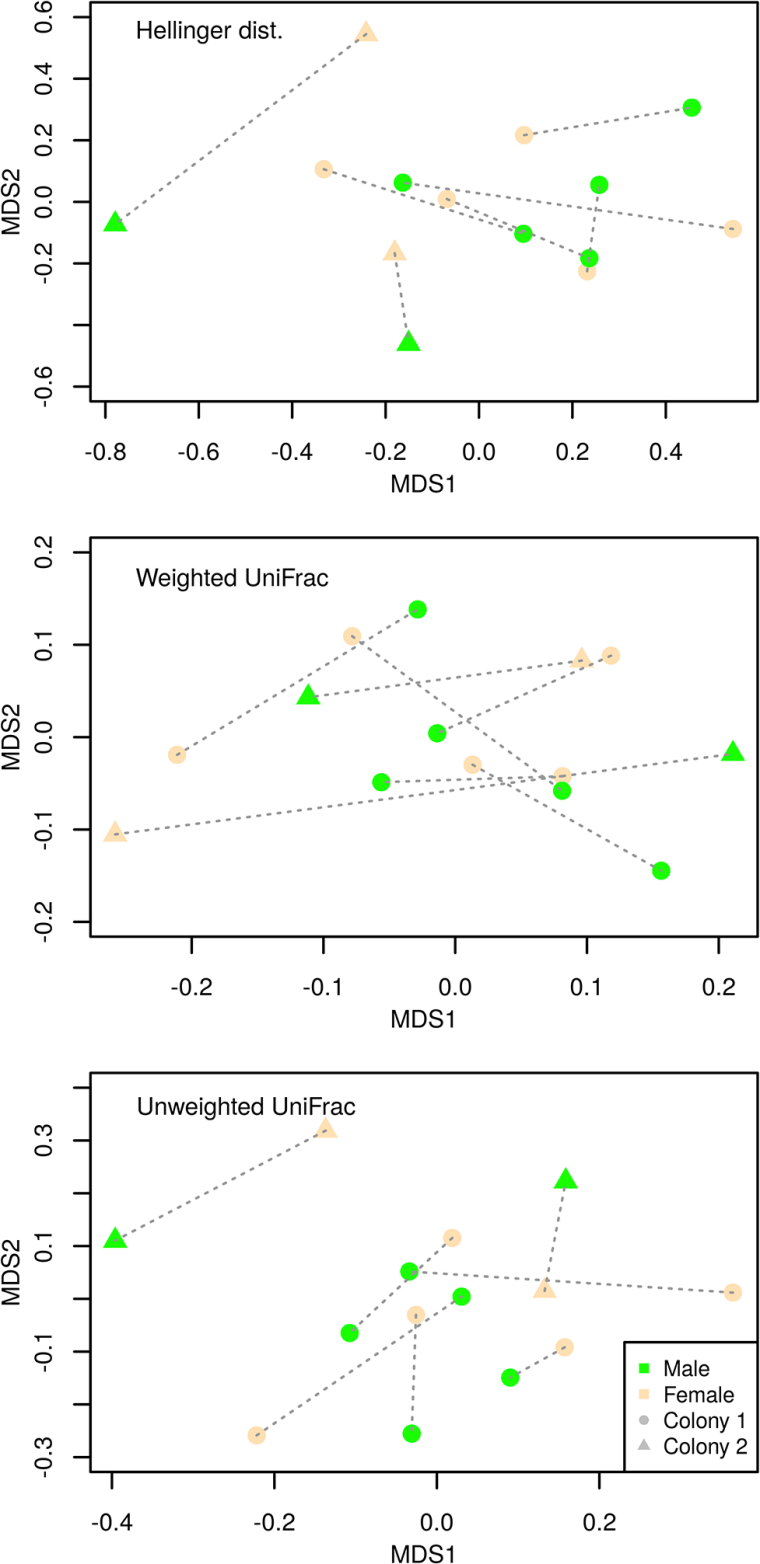
Betadiversity of barn swallow cloacal microbiome. Non-metric multidimensional scaling, based on Hellinger, unweighted and weighted UniFrac distances for barn swallow cloacal microbiota. Green and brown symbols indicate males and females, respectively. Circles and triangles correspond to the two localities sampled (Kotrbů and Šaloun, respectively). Individuals belonging to the same breeding pair are indicated by the same plotting character and connected by a dashed line.

The mean OTU number per sample, as predicted using the Chao1 index, was 179 (range = 107–424). CatchAll predictions of OTU richness were comparable with Chao1 estimates (range = 112–570 OTUs per sample, see S1 Table). The number of observed OTUs showed no variation between males vs. females (Paired t-test: t_(d.f. = 6)_ = 0.375, p = 0.721). Non-significant difference between males and females was revealed also based on other alpha-diversity indexes (p > 0.2 in all cases). Db-RDA suggested no difference in CM composition between males and females (Hellinger distances: F_(d.f. = 1,12)_ = 0.769, R^2^ = 0.055, p = 0.906, weighted UniFrac: F_(d.f. = 1,12)_ = 0.671, R^2^ = < 0.01, p = 0.672 and unweighted UniFrac: F_(d.f. = 1,12)_ = 0.977, R^2^ < 0.01, p = 0.520; Fig 2). Similarly, betadisper provided no support for differences in inter-individual CM variation between males and females (Hellinger: F_(d.f. = 1,12)_ = 0.017, p = 0.8977, weighted UniFrac: F_(d.f. = 1,12)_ = 0.176, p = 0.682 and unweighted UNIFRAC: F_(d.f. = 1,12)_ = 0.219, p = 0.648, respectively). We found no difference in proportion of individual bacterial phyla and families between males and females (Wilcoxon signed rank test: p > 0.1 and q > 0.1 in all cases). Furthermore, out of 123 OTUs with at least 0.1% high quality reads (see S2 Table), none showed any variation in relative abundance between males and females (Wilcoxon signed rank p > 0.03 and q > 0.05 in all cases). These OTUs exhibited highly aggregated distribution among sampled individuals (median value of k parameter = 0.172, inter-quartile range = 0.057–0.337, S2 Table). Db-RDAs models, based on unweighted UniFrac and Hellinger distances, suggested that individuals from different breeding colonies tended to be colonised by different bacterial OTUs (F_(d.f. = 1,12)_ = 1.357, R^2^ = 0.102, p = 0.010 and F_(d.f. = 1,12)_ = 1.618, R^2^ = 0.131, p = 0.019, respectively); however, this was largely influenced by individuals from a single breeding pair. When performing the same analysis using weighted UniFrac distances, between colony differences were not significant (F_(d.f. = 1,12)_ = 1.009, R^2^ = 0.080, p = 0.380; Fig 2).

**Fig 3:**
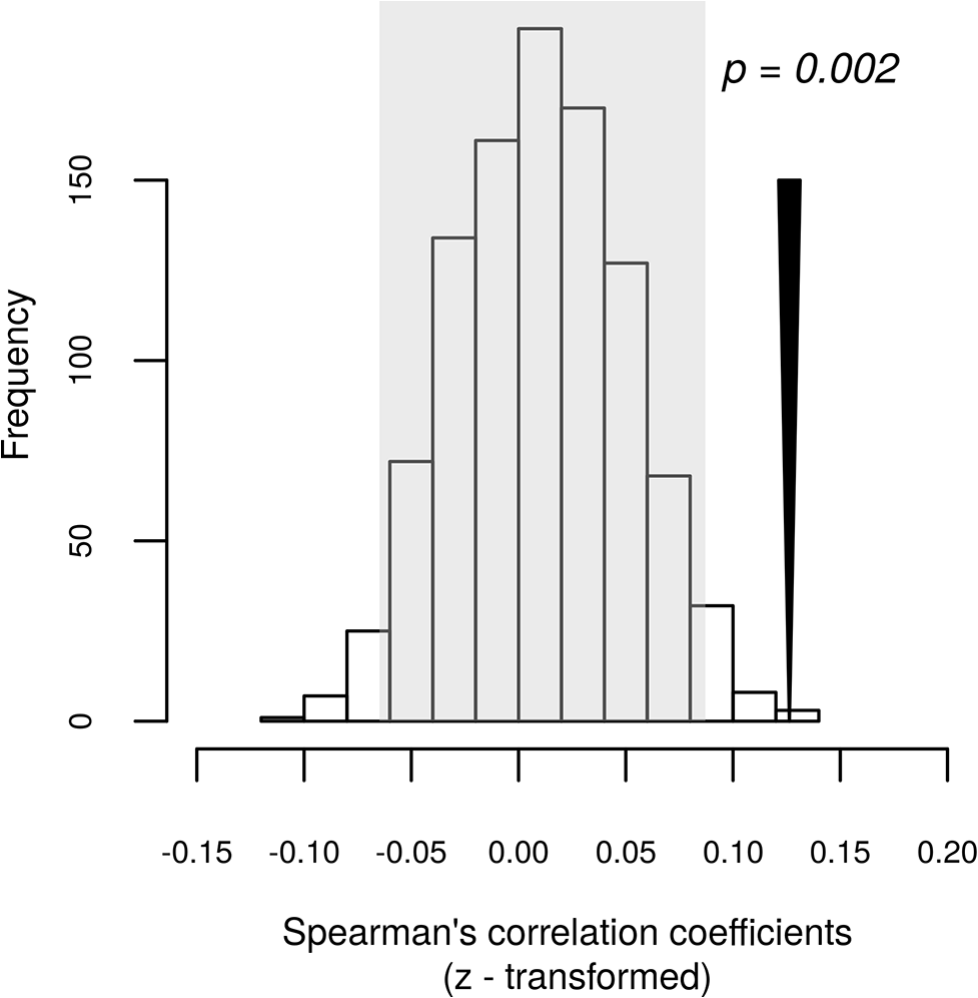
Correlation of OTU abundance between males and females within individual breeding pairs. Histogram showing the distribution of simulated means of Fisher’s z transformed Spearman’s correlation coefficient computed based on the correlation of relative abundance of individual OTUs between males and females belonging to the same breeding pair. The grey area indicates the 95% confidence interval for the simulated means. The black arrow corresponds to the mean Fisher’s z transformed Spearman’s correlation coefficient calculated based on the original community table. Permutation-based significance is indicated above the arrow.

Sample-centred permutations did not suggest a higher within-pair correlation than that expected by chance (Hellinger distances: p = 0.108, weighted UniFrac distances: p = 838 and unweighted UniFrac p = 0.220). An OTU-centred permutation model, however, indicated higher relative OTU abundance correlations between individuals in the same breeding pair than expected by chance (p = 0.002; Fig 3; standardised effect size = 2.910; untransformed mean Spearman’s r = 0.101). The result remained significant after exclusion of *Cyanobacteria* OTUs and OTUs most likely corresponding with arthropod-associated bacteria (see Discussion); i.e. not an integral part of Barn Swallow microbiota (p = 0.004, SES = 2.490, untransformed mean Spearman’s r = 0.089).

## Discussion

Barn swallow CM was dominated by species of the phyla *Proteobacteria*, *Firmucutes* and *Actinobacteria*, with a further 17 bacterial and two archaebacterial phyla recorded at low relative abundances (< 5% of high quality reads). Despite such high CM phylogenetic diversity, the community exhibited only moderate diversity at the OTU level. We detected less than 1,000 OTUs (754 non-singleton), with the number of OTUs per individual predicted using the Chao1 index ranging between 107 and 424.

To date, most research on animal-associated microbiomes has been dedicated to bacteria colonising mammalian hosts [3–5,11,15,54]. Compared to the typical GTM of most mammalian species studied thus far, barn swallow CM taxonomic composition appears to be rather distinct. The phylum *Bacteriodetes* (along with *Firmicutes*), for example, usually dominates the GTM of most mammalian species [2,11,15,55,56], but was represented by less than 5% of high quality reads in the barn swallow. On the other hand, the Phyla *Proteobacteria* and *Actinobacteria*, which were abundant in barn swallow CM, are usually under-represented in the GTM of mammalian species [55], but see [54]. Differences between mammalian GTM and barn swallow CM could conceivably be due, at least in part, to the distal position of the cloaca in the digestive tract and its intermittent connection with the urogenital tract and the external environment. Our recent data, however, have shown no pronounced difference between CM and GTM community structure in passerine birds (Kropackova, *unpublished results*). Furthermore, a number of recent high-throughput sequencing studies have also shown bird GTM to be dominated by *Proteobacteria*, *Actinobacteria* and *Firmicutes* [57–61].

At a lower taxonomic level, many genera dominating in CM, such as *Enterobacter*, *Streptococcus*, *Enterococcus*, *Clostridium*, *Lactobacillus*, *Lactococcus*, *Turicibacter* and members of the *Ruminococcaceae* family, are facultative symbionts or commensals inhabiting the digestive tracts of many vertebrate species [2,56,62]. We also detected several OTUs, such as *Staphylococcus*, *Janthinobacterium*, *Corynebacterium*, *Aerococcus* and *Brevibacterium*, that commonly colonise the skin’s surface [63] and OTUs associated with other parts of the animal’s body, such as *Rothia*, *Porphyromonas Enhydrobacter* and *Actinobacillus*, see for example [64]. Finally, barn swallow CM composition may also partly reflect the bird’s diet, which is composed of flying insects and other arthropods present in aerial plankton. Several abundant OTUs, including *Hamiltonella*, *Rickettsiella* and *Wohlfahrtiimonas*, correspond to symbiotic or pathogenic bacteria of arthropods [65–67]. Their widespread presence in barn swallow CM, therefore, is most probably a consequence of its foraging specialisation.

High inter-individual variation appears to be a general feature of the core mammalian GTM microbiome [12,19,68,69], but see [70]. This also appears to be true for barn swallow CM, with most OTUs detected in a single individual only and a relatively low proportion detected in more than 50% of individuals. Rarefaction analysis suggests this level of inter-individual heterogeneity is unlikely to be an artefact caused by insufficient sequencing depth. High interindividual variation in OTU presence vs. absence was further underscored by low values of k parameter, indicating highly aggregated OTU distribution among sampled hosts. This is comparable with the aggregation pattern observed in vertebrate macroparasites [51].The barn swallow is a trans-Saharan migrant spending more than half-a-year outside its breeding locality [71]; hence we speculate that high inter-individual CM variation may be shaped, to some extent, by the heterogeneity of biotic and abiotic factors over the migration and wintering periods. If tso, the CM could be viewed as a 'carry-over' effect that might contribute to variation in reproductive output over the breeding season [72]. Interestingly, NMDS and db-RDA indicated no pronounced difference in CM in individuals from different breeding colonies. Although a larger sample size would be desirable for a more robust conclusion, this result suggests that variation in environmental conditions operating at small spatial scales during the breeding season has a limited effect on CM composition.

It has previously been shown that animal-associated microbiome composition is correlated with physiological stress [73], hormonal status [74], reproduction [75] and metabolic rate [4]. In barn swallows, there is a pronounced sexual difference in parental care investment [76], along with overall physiological and hormonal status [77,78], over the breeding season. Nevertheless, our data suggest that these aspects are not associated with systematic differences in CM between males and females, with neither dominant OTU abundance nor CM taxonomic composition exhibiting any apparent sex-dependent variation. Furthermore, both CM alpha (i.e. OTU richness) and beta diversity (i.e. level of inter-individual variation) were comparable between males and females, which is consistent with recent work on New World vultures [79]

Previous experimental and correlative studies have demonstrated cloacal contact during within-pair copulation to be an important factor shaping CM composition and contributing to CM similarity between individuals of the same breeding pair [25,30,31]. At the same time, CM composition has been suggested to have an important influence on within- and extra-pair mate choice and propensity to copulation in general, as both beneficial and potentially pathogenic bacteria may be transmitted during copulation [17,18,22,23,31]. Indeed, permutation-based analysis of barn swallow CM suggests that OTU abundance is correlated between individuals of the same breeding pair. The effect-size of this pattern is rather small, however, which is consistent with NMDS ordination and, more explicitly, with resampling tests based on between-sample distances, which show that CM similarities within breeding pairs were not lower than expected by chance. It is possible that within-pair similarities in CM may be, at least partly, jammed by CM transfer during extra-pair copulations, which occur frequently in the study populations [29]. In addition, it is worth mentioning that samples were collected approx. 2–3 weeks after egg fetilization. Recent manipulative study of White et al. [25] showed that similarity of CM community between social partners in kittiwakes (*Rissa tridactyla)* decrease rapidly after experimental prevention of copulations. Consistent with this observation, our data indicate that the potential for sexually transmitted bacteria to result in a major long-term CM shift in barn swallow is rather low.

## Supporting Information

S1 Fig Rarefaction analysis. Rarefaction curves for the number of 97% OTUs detected in individual samples according to sequencing depth. Calculations were based on 10 sub-sampled datasets for each sequencing depth (0–3000 randomly selected sequences). Colours correspond to individual breeding pairs. Males and females are indicated by triangles and circles, respectively.
(JPG)

S2 Fig Taxonomic classification of barn swallow cloacal microbiota. Barplots showing taxonomic assignment (based on RDP classifier; 80% confidence threshold) of 454 sequences to A) Phylum and B) Class level for sequences corresponding to the five most abundant phyla (represented by *Proteobacteria*, *Firmicutes*, *Actinobacteria*, *Tenericutes* and *Bacteroidetes*). This subset accounts for ca. 87% of high quality sequences generated during this study. Facets (A-H) correspond to individual breeding pairs. Samples within facets are sorted according to sexual identity (F = females, M = males). Detailed taxonomic classification of the dominant OTUs is provided in S2 Table.
(JPG)

S1 Table Details on samples used in this study. (XLS)

S2 Table List of dominant OTUs detected in the barn swallow cloacal microbiome. Shown are OTUs represented by > 0.1% sequences and detected in at least two individuals. The Table includes information on taxonomic classification to genus level, proportion of high quality reads represented by a given OTU (Prop. Seqs.), the proportion of individuals for which a given OTU was detected (Prop. Individual) and the corrected moment estimate of k of the negative binomial distribution (k param.).
(XLS)
