# Supplementary figures and images for: Cloacal Microbiome Structure in a Long-Distance Migratory Bird Assessed Using Deep 16sRNA Pyrosequencing
Source: PLoS One. 2015 Sep 11;10(9):e0137401. doi: 10.1371/journal.pone.0137401 (PMC4567286; doi:10.1371/journal.pone.0137401)

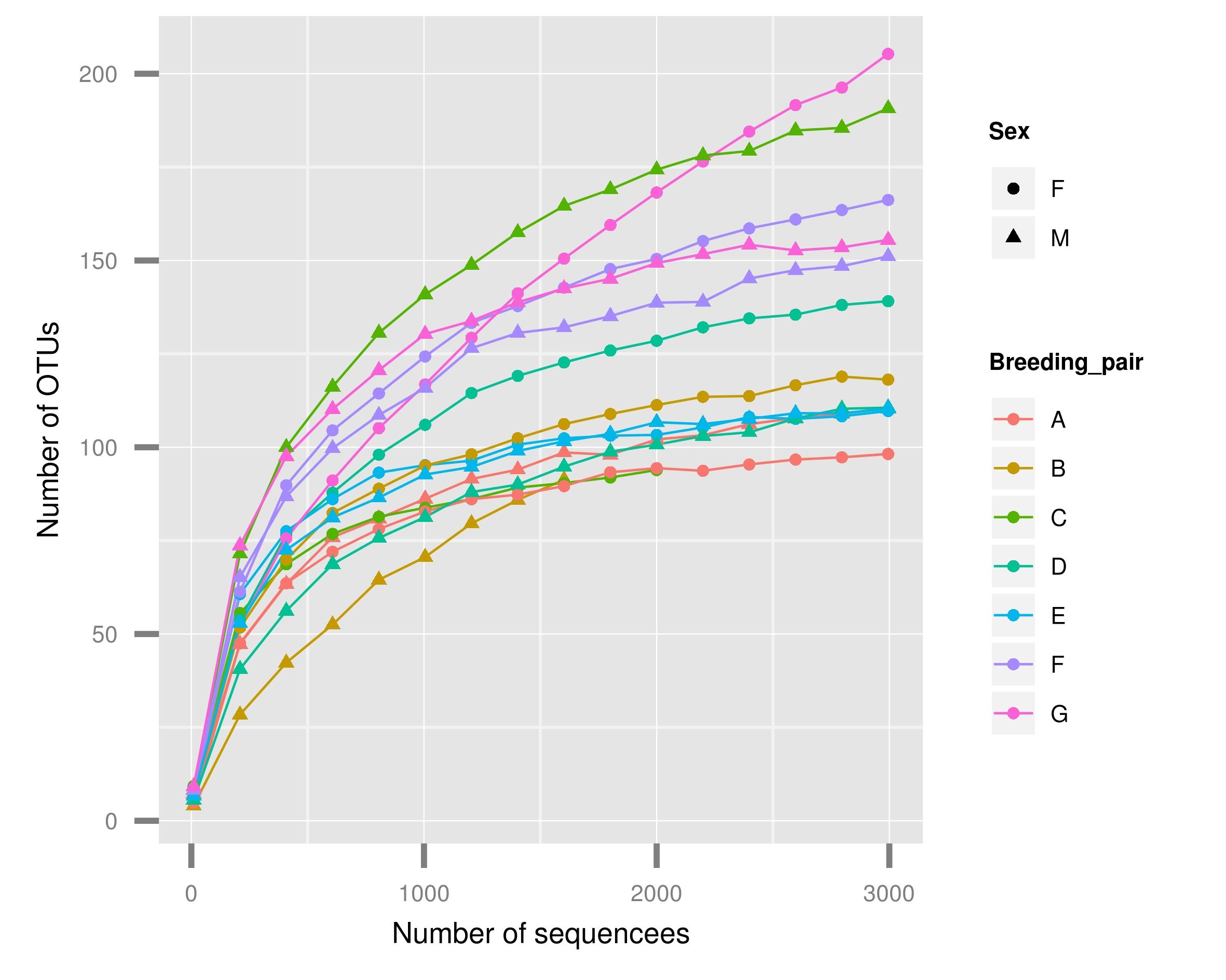

Supplement: S1 Fig — Rarefaction curves for the number of 97% OTUs detected in individual samples according to sequencing depth. Calculations were based on 10 sub-sampled datasets for each sequencing depth (0–3000 randomly selected sequences). Colours correspond to individual breeding pairs. Males and females are indicated by triangles and circles, respectively. (JPG) [file pone.0137401.s001.jpg]

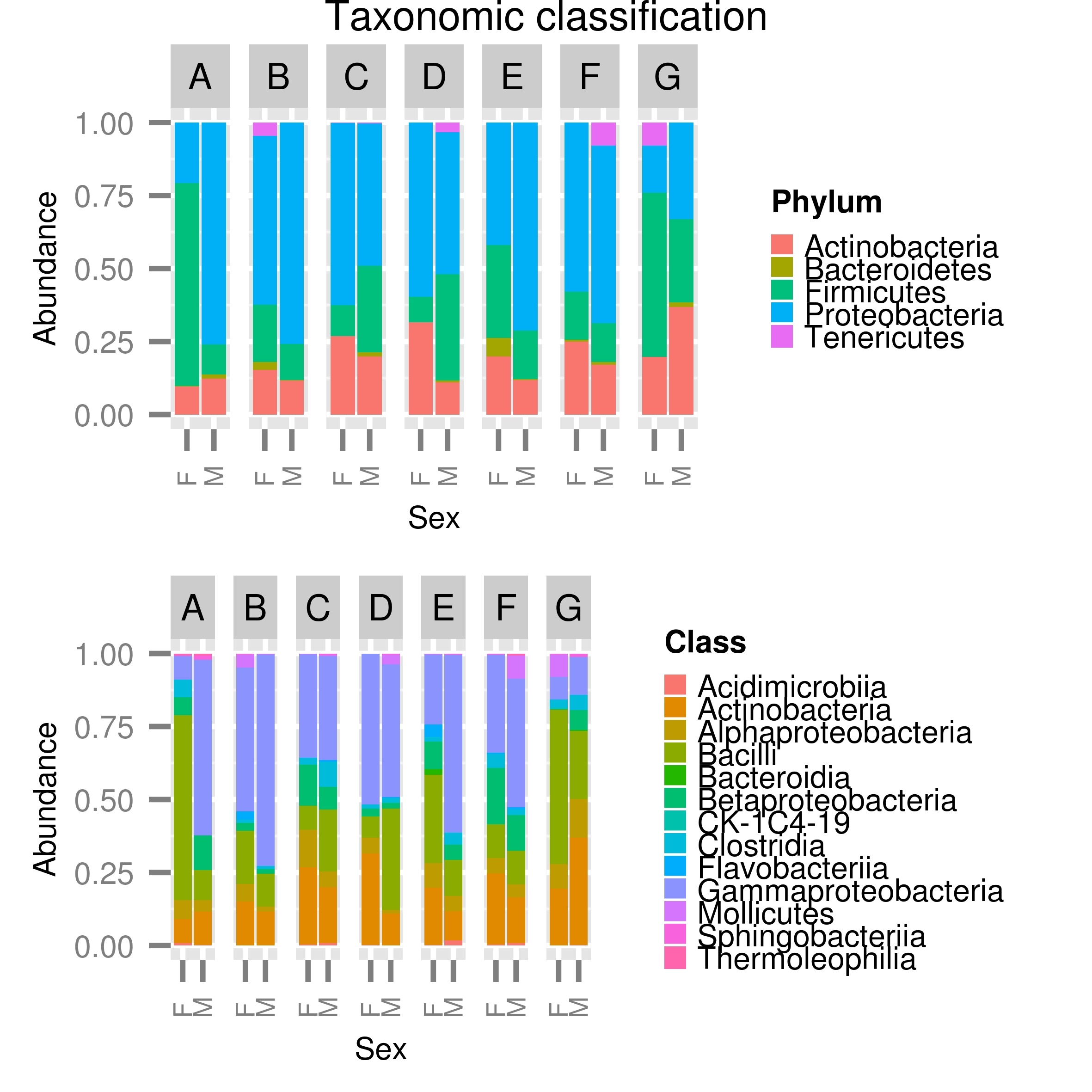

Supplement: S2 Fig — Barplots showing taxonomic assignment (based on RDP classifier; 80% confidence threshold) of 454 sequences to A) Phylum and B) Class level for sequences corresponding to the five most abundant phyla (represented by Proteobacteria, Firmicutes, Actinobacteria, Tenericutes and Bacteroidetes). This subset accounts for ca. 87% of high quality sequences generated during this study. Facets (A-H) correspond to individual breeding pairs. Samples within facets are sorted according to sexual identity (F = females, M = males). Detailed taxonomic classification of the dominant OTUs is provided in S2 Table. (JPG) [file pone.0137401.s002.jpg]
